# Suboptimal controller design of global active noise control system for various acoustic environments

**DOI:** 10.1038/s41598-023-32261-9

**Published:** 2023-04-03

**Authors:** Ikchae Jeong, Youngjin Park

**Affiliations:** Department of Mechanical Engineering, Korea Advanced Institute of Science and Technology, Daejeon, 34141 Korea

**Keywords:** Electrical and electronic engineering, Mechanical engineering

## Abstract

Conventional active noise control (ANC) systems in enclosed spaces are not easy to implement experimentally because they require a large number of microphones to measure sound pressure in global areas. Even if such systems are possible, if there are any changes in the locations of noise sources or surrounding objects, or if ANC system moves to another enclosed space, an expensive and time-consuming experimental calibration is again required. Implementation of global ANC in enclosed spaces is thus difficult. Therefore, we designed a global ANC system that can be used in various acoustic environments. The main idea involves suboptimal open-loop controller design in the free field. By using an open-loop controller, a controller calibrated once can be used in various acoustic environments. A controller designed in the free field derive a suboptimal solution without bias toward a specific acoustic environment. For controller design in the free field, we propose an experimental calibration approach in which the arrangement and the number of control speakers and microphones are determined by the frequency range and radiation pattern of the noise source. We conducted simulations and experiments to show that the designed controller in the free field is sufficiently effective in other enclosed spaces.

## Introduction

Active noise control (ANC) is a method to reduce unwanted noise by generating opposite-phase sound. ANC can be divided into local and global ANC methods according to the control area. Most ANC studies have considered local ANC, in which the control area is limited around a point like an error microphone^1–3^. Global ANC takes the entire space as the control area. Global ANC can be divided into two cases, in which the noise source is either inside the control area or outside it. When the noise source is outside, most noise flows through the window; studies have looked at active windows to reduce noise entering in this way^4–6^. In this paper, we focus on a noise source inside the control area. To control the global area of the enclosed space, the objective function must be set as acoustic potential energy in the control area^7,8^. To calculate this energy, the challenge is to measure the sound pressure of the whole space, which must be approximated as the sum of the squared sound pressures at evenly distributed measurement points; however, many microphones are still required for the accuracy of approximation^9^. Of course, there have also been attempts to implement global ANC with modal characteristics using a small number of microphones^10–17^; these attempts have encountered difficulties, however, in general cases, because methods are only effective at frequencies lower than the Schroeder frequency^18^. Therefore, many microphones are still required in the general situation. In addition, if the acoustic environment changes, such as changes in locations of noise sources or surrounding objects, or movements of the ANC system to other enclosed spaces, existing solutions cannot be used, and expensive and time-consuming experimental calibration is required again. This inhibits general applications of global ANC. In addition, there have been various attempts for global ANC in an enclosed space^19–22^. However, all studies focus only on a specific enclosed space, and using them in various enclosed spaces is difficult. Then our research objective is thus to design a global ANC controller for various acoustic environments, even though the performance of this controller might be suboptimal.

The main idea is the design of a suboptimal open-loop controller in a free field. An open-loop controller can be implemented without additional calibration whenever the acoustic environment changes. A controller design through the collocation of control speakers that radiate a sound field of opposite phase to the noise source in the free field can lead to suboptimal solutions that are not biased toward a certain acoustic environment. A suboptimal controller has the advantage that it can be used in various acoustic environments without additional calibration, even though its control performance may be slightly lower than that of the optimal controller for each acoustic environment. The controller design can be achieved by minimizing the acoustic power radiated by the noise sources and control speakers from the fact that acoustic power is directly proportional to acoustic potential energy in the free field^23^. Since the possibility of this method was mentioned by Kempton^24^, there have been studies that have theoretically developed and experimentally attempted this using Kirchhoff's theorem^25–27^. However, since the method using Kirchhoff's theorem requires a continuous control source, practical methods using discrete control sources have been studied^28–34^. Afterwards, representative theoretical researches were performed on global ANC in the free field using collocation of noise and control sources^35,36^. However, there are limits to experimental implementation because previous analyses were performed in the frequency domain using ideal monopoles. Similarly, studies have been performed using ASAC (active structural acoustic control), in which global ANC was induced by vibration of a plate induced by casing the noise source with an additional plate^37–40^. The collocation method mentioned above was used only in the free field, and we intend to apply it to the enclosed space.

Also, the previous experimental implementations set configurations of control speakers (shakers) and microphones without specific standards. So, estimation of the objective function may thus not be accurate and controllers will be biased to specific acoustic environments, and difficult to use in other acoustic environments. Also, control speakers and error microphones placed without specific standards may cause spatial aliasing, leading to performance degradation of sound field estimation and control^41,42^. To avoid this problem, a configuration of the control speakers and error microphones was determined by referencing studies in the fields of array signal processing and sound field reproduction^43–48^. Finally, the configuration of the control speakers and error microphones was set according to the radiation pattern and frequency range of the noise source. Once configured, the controller was calibrated using the open-loop ANC algorithm, which minimizes the acoustic power. Simulation and experiments were conducted to verify that the suboptimal controller was effective in various enclosed spaces.

## Methods

### Configuration method of control speakers and error microphones

The ANC system consists of a reference sensor, control speaker, and error microphone^49^. We are not focusing on a specific noise source because the goal is to verify the feasibility of the proposed method regardless of the noise source. The reference sensor's type and configuration depend on the noise source's characteristics. Therefore, we will not cover the type or configuration method of the reference sensor in this paper. The loudspeaker with a freely configurable frequency range from which the reference signal (speaker input signal) can be determined and obtained accurately was used as the noise source. Through this, the maximum control performance of the proposed method will be confirmed regardless of the reference sensor. In this paper, the frequency range of interest is set from 170 to 378 Hz arbitrarily. In practice, given a noise source to be controlled, a frequency range of interest is determined according to the spectrum of the noise source. Accordingly, the configuration of control speakers and error microphones can be determined. In spatial control, like global ANC, discrete placement of the control speakers and error microphones can cause spatial aliasing, which affects the controllable and measurable frequency range and the radiation pattern. So, we would determine the configuration of control speakers and error microphones so that spatial aliasing does not occur.

First, we were only interested in outgoing waves, so we set the radius of the microphone array same or larger than half the wavelength at which about 95 percent reduces the effects of evanescent waves^43^.

1 $$\frac{\uplambda }{2}\le r$$

Here, $\uplambda$ is the wavelength and $r$ is the radius of the microphone array. Measurable radiation patterns and a sampling method determined the arrangement and number of microphones. We used a spherical harmonic function as the basis of the radiation pattern. This function has already been used in 3D sound field reproduction, like ambisonics^50^. The spherical harmonics degree indicates the complexity of the radiation pattern. Measurable spherical harmonics degree without spatial aliasing is determined by the radius of the microphone array and the upper frequency limit^43^.

2 $$kr<N$$

Here, $k$ is the wave number and $N$ is the measurable spherical harmonics degree. Once the measurable degree was determined, the number and arrangement of microphones were determined by the sampling method. Typical sampling methods include uniform sampling, equal-angle sampling, and Gaussian sampling^43^. We decided to use the Gaussian sampling method because it allows an easy experimental setup. The Gaussian sampling method requires a ${2(N+1)}^{2}$ number of microphones. The sound field of the noise source can be measured through this microphone arrangement; the maximum degree of spherical harmonics of the noise source can be estimated through spherical harmonics expansion^45^. We can also measure the acoustic power with this arrangement. Because we use an open-loop algorithm, the determined microphone array is used in measurement to calculate the fixed control filter and is not used during actual ANC.

To make it easy to quantify the spatial aliasing issues and to control the 3-D space equally, we decided to place the control speakers at the vertexes of a platonic solid^46,47^. The upper limit of the radius was determined by the spatial aliasing frequency^48^.

3 $$f\le \frac{\mathrm{c}}{2\mathrm{a}}$$

Here, $f$ is the controllable frequency, $\mathrm{c}$ is the speed of sound, and $\mathrm{a}$ is the radius of the speaker array. This is the same argument mentioned by Nelson et al.^35^. Number of control speakers was decided according to the noise source’s maximum degree of spherical harmonics^46^. For effective control of spherical harmonics, there must be at least as many control speakers as the basis of spherical harmonics to be controlled.

4 $${({N}_{M}+1)}^{2}\le K$$

**Figure 1:**
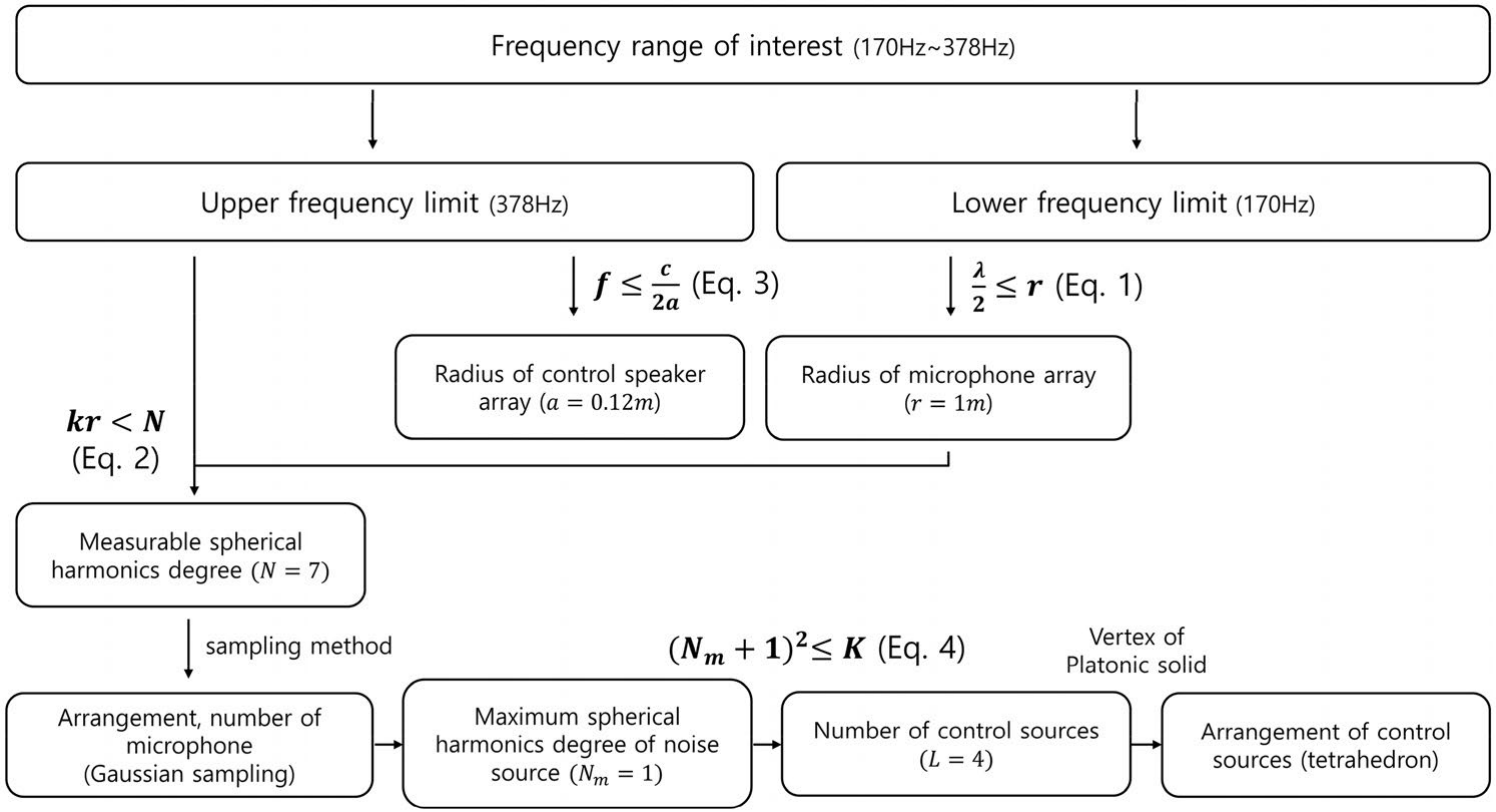
Flow chart of configuration method of control speakers and microphones. Once the frequency range of interest was determined by the spectrum of the noise source, the placement and number of control speakers and microphones can be determined according to the lower and upper frequency limits. Here, $f$ is the frequency, $c$ is the sound speed, $a$ is the radius of the control speaker array, $\uplambda$ is the wavelength, $r$ is the radius of the microphone array, $k$ is the wavenumber, $N$ is the measurable spherical harmonics degree, ${N}_{m}$ is the maximum spherical harmonics degree of the noise source, $K$ is the number of control speakers.

Here, ${N}_{M}$ is the noise source’s maximum degree of spherical harmonics and $K$ is the number of control speakers. Finally, by combining all of the above, we can determine the location and number of control speakers and error microphones according to the frequency range of interest. Figure 1 shows the step sequence.

### ANC algorithm

**Figure 2:**
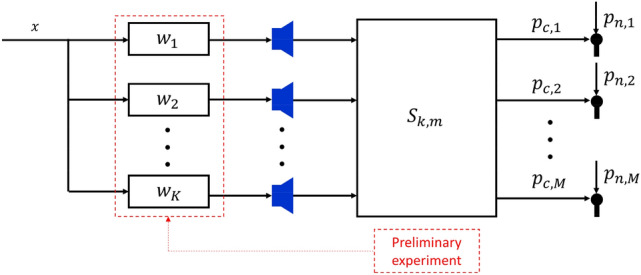
Flow chart of open-loop acoustic power minimization algorithm. Since a large number of microphones are required for acoustic power measurements, an open-loop ANC algorithm, which does not require a microphone during ANC, is used. The control filter is calculated by a preliminary experiment that minimizes acoustic power in anechoic chamber. Microphones are placed on a spherical surface surrounding the noise source. Here, $x$ is the reference signal, ${w}_{k}$ is the control filter of ${k}{th}$ control speaker, and ${S}_{k,m}$ is the secondary path from the ${k}{th}$ control speaker to the ${m}{th}$ microphone. ${p}_{n,m}$ is the sound pressure by the noise source at the ${m}{th}$ microphone and ${p}_{c,m}$ is the sound pressure by control speakers at the ${m}{th}$ error microphone.

Most ANC algorithms use the adaptive filtering method updated by signals measured by error microphones in the control area. A large number of microphones are required to measure the acoustic power, which is impractical. As such, we decided to use the open-loop ANC algorithm, which does not use error microphones during ANC. With an open-loop algorithm, a controller calibrated once can be used in various acoustic environments. For the algorithm’s stability, the control filter is designed as an FIR structure. The filter length was set to 512 taps to express the tale of the impulse response sufficiently. Using the multi-channel Wiener filter solution^51^, we calculated the control filter through a preliminary experiment in an anechoic chamber. Figure 2 provides a flow chart of the ANC algorithm. Equation (5) shows the objective function.

5 $$J=\sum_{m=1}^{M}E\left[{\left|{p}_{n,m}(n)+{p}_{c,m}(n)\right|}^{2}\right]$$

6 $${p}_{c,m}(n)=\sum_{k=1}^{K}\sum_{l=0}^{L-1}{w}_{k}\left(l\right){{x}^{^{\prime}}}_{k,m}(n-l)$$

Here, $J$ is the objective function, $M$ is the number of microphones, ${p}_{n,m}$ is the sound pressure from the noise source at the ${m}{th}$ microphone, ${p}_{c,m}$ is the sound pressure from the control speaker at the ${m}{th}$ microphone, $E[\cdot ]$ is the expected value, $K$ is the number of control speakers, ${w}_{k}$ is a control filter of the ${k}{th}$ control speaker, $L$ is the length of the control filter, and ${{x}^{^{\prime}}}_{k,m}$ is the reference signal filtered by ${S}_{k,m}$, the secondary path from the ${k}{th}$ control speaker to the ${m}{th}$ microphone. Wiener filter that minimizes the cost function satisfies the following partial differential equation which is called the orthogonality principle or projection theorem.

7 $$\frac{\partial J}{\partial {{w}_{h}}^{*}(r)}=-2\sum_{m=1}^{M}E\left[\left\{{p}_{n,m}\left(n\right)+{p}_{c,m}\left(n\right)\right\}{{{x}^{^{\prime}}}_{h,m}}^{*}\left(n-r\right)\right]=0$$

where, $r$ is index of filter value and $r=0, 1, \dots , L-1$, $h=1, 2, \dots , K$. Equation (7) can be summarized as below.

8 $$\sum_{m=1}^{M}-E\left[{p}_{n,m}\left(n\right){{{x}^{^{\prime}}}_{h,m}}^{*}\left(n-r\right)\right]=\sum_{m=1}^{M}\sum_{k=1}^{K}\sum_{l=0}^{L-1}{w}_{k}\left(l\right)E\left[{{x}^{^{\prime}}}_{k,m}\left(n-l\right){{{x}^{^{\prime}}}_{h,m}}^{*}\left(n-r\right)\right]$$

let, $-E\left[{p}_{n,m}\left(n\right){{{x}^{^{\prime}}}_{h,m}}^{*}\left(n-r\right)\right]={P}_{m,h}(r)$, cross correlation of ${{x}^{^{\prime}}}_{h,m}$ and ${p}_{n,m}$. $E\left[{{x}^{^{\prime}}}_{k,m}\left(n-l\right){{{x}^{^{\prime}}}_{h,m}}^{*}\left(n-r\right)\right]={R}_{m,kh}(r-l)$, cross correlation of ${{x}^{^{\prime}}}_{k,m}$ and ${{x}^{^{\prime}}}_{h,m}$. Equation (8) can be summarized as belows.

9 $${A}_{n,h}(r)=\sum_{k=1}^{K}\sum_{l=0}^{L-1}{w}_{k}\left(l\right){A}_{c,kh}(r-l)$$

10 $${A}_{n,h}(r)=\sum_{m=1}^{M}{P}_{m,h}(r)$$

11 $${A}_{c,kh}(r-l)=\sum_{m=1}^{M}{R}_{m,kh}(r-l)$$

By summarizing this equation in a matrix form, we can get optimal filter value that minimizes the cost function.

12 $${\varvec{W}}={{{\varvec{A}}}_{{\varvec{C}}}}^{-1}{{\varvec{A}}}_{{\varvec{N}}}$$

13 $${\varvec{W}}={\left[\begin{array}{cc}\begin{array}{cc}{{{\varvec{w}}}_{1}}^{{\varvec{T}}}& {{{\varvec{w}}}_{2}}^{{\varvec{T}}}\end{array}& \begin{array}{cc}\cdots & {{{\varvec{w}}}_{{\varvec{K}}}}^{{\varvec{T}}}\end{array}\end{array}\right]}^{{\varvec{T}}}$$

14 $${{\varvec{w}}}_{{\varvec{k}}}={\left[\begin{array}{cc}\begin{array}{cc}{w}_{k}(0)& {w}_{k}(1)\end{array}& \begin{array}{cc}\cdots & {w}_{k}(L-1)\end{array}\end{array}\right]}^{T}$$

15 $${{\varvec{A}}}_{{\varvec{C}}}=\left[\begin{array}{cc}\begin{array}{cc}{{\varvec{A}}}_{{{\varvec{C}}}_{11}}& {{\varvec{A}}}_{{{\varvec{C}}}_{12}}\\ {{\varvec{A}}}_{{{\varvec{C}}}_{21}}& {{\varvec{A}}}_{{{\varvec{C}}}_{22}}\end{array}& \begin{array}{cc}\cdots & {{\varvec{A}}}_{{{\varvec{C}}}_{1{\varvec{K}}}}\\ \cdots & {{\varvec{A}}}_{{{\varvec{C}}}_{2{\varvec{K}}}}\end{array}\\ \begin{array}{cc}\vdots & \vdots \\ {{\varvec{A}}}_{{{\varvec{C}}}_{{\varvec{K}}1}}& {{\varvec{A}}}_{{{\varvec{C}}}_{\varvec{K}2}}\end{array}& \begin{array}{cc}\ddots & \vdots \\ \cdots & {{\varvec{A}}}_{{{\varvec{C}}}_{{\varvec{K}}{\varvec{K}}}}\end{array}\end{array}\right]$$

16 $${{\varvec{A}}}_{{{\varvec{C}}}_{{\varvec{k}}{\varvec{h}}}}=\left[\begin{array}{cc}\begin{array}{cc}{A}_{c,kh}\left(0\right)& {A}_{c,kh}\left(-1\right)\\ {A}_{c,kh}\left(1\right)& {A}_{c,kh}\left(0\right)\end{array}& \begin{array}{cc}\cdots & {A}_{c,kh}\left(-L+1\right)\\ \cdots & {A}_{c,kh}\left(-L+2\right)\end{array}\\ \begin{array}{cc}\vdots & \vdots \\ {A}_{c,kh}\left(L-1\right)& {A}_{c,kh}\left(L-2\right)\end{array}& \begin{array}{cc}\ddots & \vdots \\ \cdots & {A}_{c,kh}\left(0\right)\end{array}\end{array}\right]$$

17 $${{\varvec{A}}}_{{\varvec{N}}}={\left[\begin{array}{cc}\begin{array}{cc}{{{\varvec{A}}}_{{{\varvec{N}}}_{1}}}^{{\varvec{T}}}& {{{\varvec{A}}}_{{{\varvec{N}}}_{2}}}^{{\varvec{T}}}\end{array}& \begin{array}{cc}\cdots & {{{\varvec{A}}}_{{{\varvec{N}}}_{{\varvec{K}}}}}^{{\varvec{T}}}\end{array}\end{array}\right]}^{{\varvec{T}}}$$

18 $${{\varvec{A}}}_{{{\varvec{N}}}_{{\varvec{h}}}}={\left[\begin{array}{cc}\begin{array}{cc}{A}_{n,h}(0)& {A}_{n,h}(1)\end{array}& \begin{array}{cc}\cdots & {A}_{n,h}(L-1)\end{array}\end{array}\right]}^{{\varvec{T}}}$$

## Results

### Simulation

**Figure 3:**
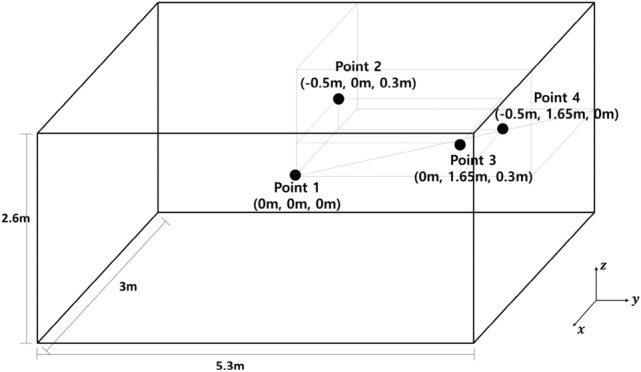
Candidates of noise source location in enclosed space.

A simulation was conducted to verify the feasibility that the controller designed in a free field could be a suboptimal solution in an enclosed space. In the simulation, performances of the two controllers, one designed in a free field (proposed method) and the other in an enclosed space, were compared in the frequency domain using FEM simulation in COMSOL. A rectangular cavity with brick wall properties of 5.3 m $\times$ 3 m $\times$ 2.6 m (Width $\times$ Depth $\times$ Height) was used as the enclosed space. A single monopole was used as a noise source, and a single monopole control source was positioned 0.1 m above the noise source on the z-axis. The simulation was conducted for a noise of 500 Hz single frequency. The controller in the free field was designed to minimize the acoustic power; the controller in the enclosed space was designed to minimize the acoustic potential energy when the noise source is at the center point (0 m, 0 m, 0 m). The performance of the two controllers was compared depending on the location of the noise source. Point 1 (0 m, 0 m, 0 m), point 2 (− 0.5 m, 0 m, 0.3 m), point 3 (0 m, 1.65 m, 0.3 m), and point 4 (− 0.5 m, 1.65 m, 0 m) are candidates of the noise source location. Figure 3 shows the candidate of the noise source location.

Of course, control source locations are point 1 (0 m, 0 m, 0.1 m), point 2 (− 0.5 m, 0 m, 0.4 m), point 3 (0 m, 1.65 m, 0.4 m), and point 4 (− 0.5 m, 1.65 m, 0.1 m) at each case. Candidates of the noise source were selected considering the symmetricity based on the center point. Then, noise reduction was calculated based on the acoustic potential energy in the enclosed space.

19 $$NR(noise \ reduction)=10{\mathrm{log}}_{10}\frac{{\int }_{V}{\left|{P}_{n,500Hz}\right|}^{2}dV}{{\int }_{V}{\left|{E}_{500Hz}\right|}^{2}dV}$$

where, ${P}_{n,500Hz}, {E}_{500Hz}$ is the complex sound pressure at 500 Hz by noise source before and after control, respectively. $V$ is the volume of the enclosed space. Table 1 shows the noise reductions of the controller designed in the free field and enclosed space when the noise source is located at points 1, 2, 3, and 4.

**Table 1:** Noise reduction of the controllers designed in the free field to minimize the acoustic power and in the enclosed space to minimize the acoustic potential energy when the noise source is located at the center point, depending on the location of the noise source.

Noise source location	Point 1	Point 2	Point 3	Point 4
Free field controller	8.2 dB	11.9 dB	16.0 dB	13.9 dB
Enclosed space controller	9.0 dB	9.1 dB	13.6 dB	11.6 dB

### Experiment

**Figure 4:**
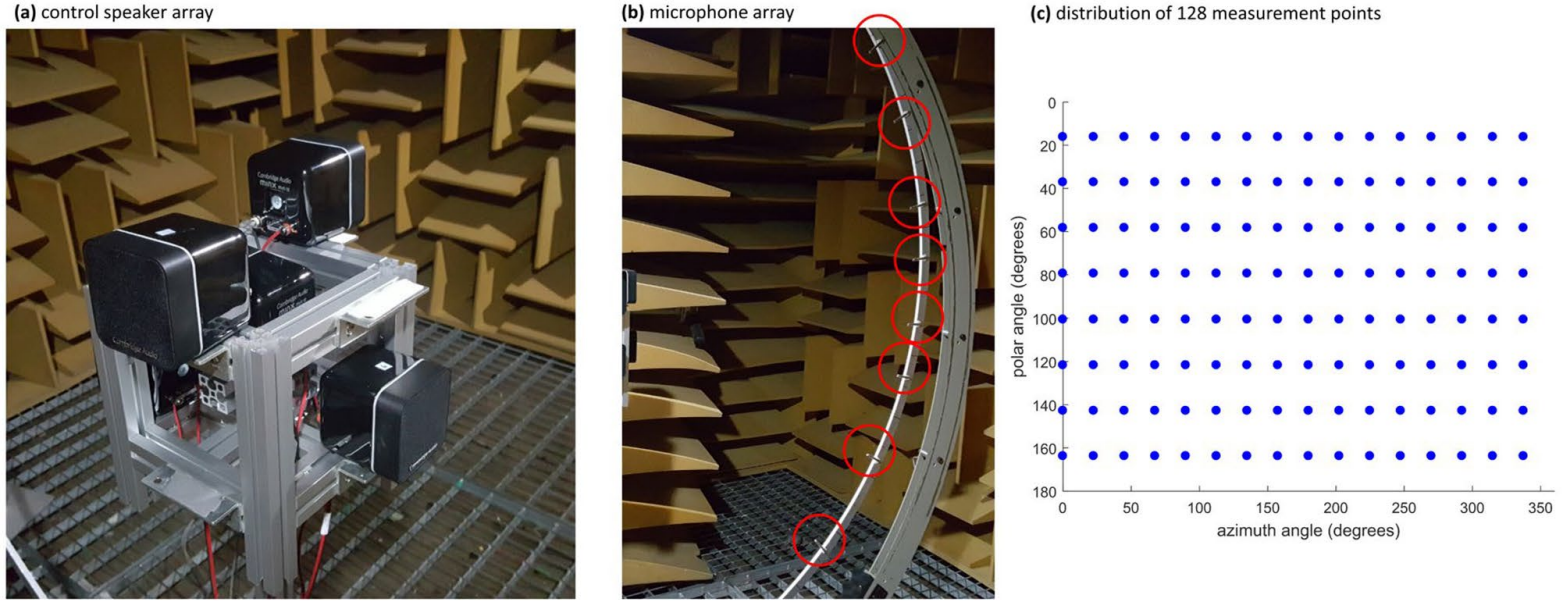
The experimental setting for controller design in an anechoic chamber and distribution of 128 measurement points. (**a**) Control speaker array; (**b**) Microphone array; (**c**) Distribution of 128 measurement points. Four control speakers were placed near the noise source at the vertex of a regular tetrahedron. Eight microphones (red circles) were rotated to measure acoustic power at a total 128 measurement points according to Gaussian sampling method. All 128 measurement points are 1 m away from the noise source.

The experiment consists of two steps. The first step is controller design in the free field using the proposed ANC algorithm and configuration method of control speakers and error microphones. The second step is the global ANC experiment with the controller obtained in the first step. Commercial speakers were used for both the noise source and control speakers, and we used B&K microphones (type 4190). DS1103 model from Dspace was used as a digital signal processor. Control speakers and error microphones were arranged based on the frequency range of interest with the method mentioned in the Methods section. Figure 4a provides a picture of the control speaker array. Four control speakers were placed at the vertexes of a tetrahedron. The radius of the speaker array was 0.12 m. Figure 4b provides a picture of the microphone array. Positions of microphones were determined by the Gaussian sampling method. The sound field was measured at a total of 128 points while rotating eight microphones located on a semicircle. The radius of the microphone array was 1 m. Figure 4c shows the distribution of 128 measurement points depending on the azimuth angle and polar angle.

**Figure 5:**
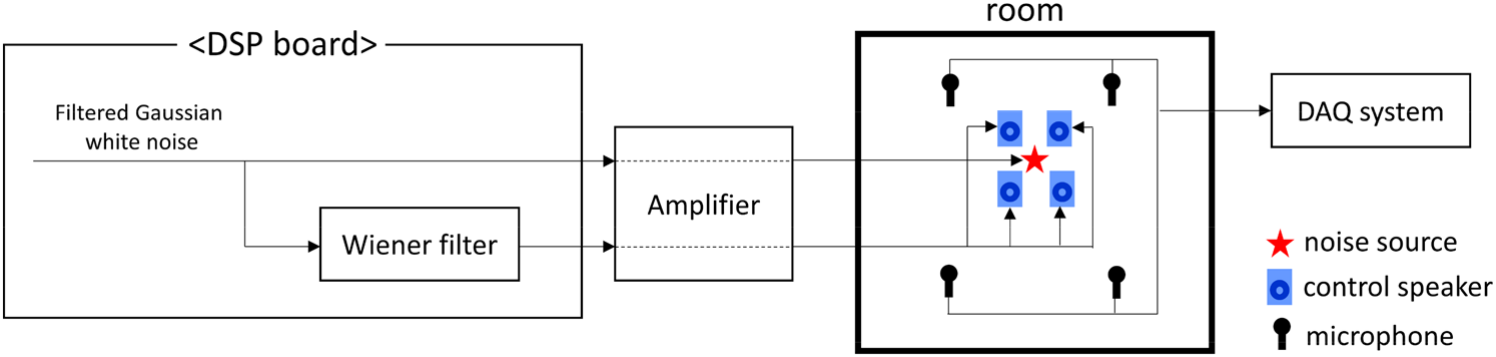
Schematic diagram of global active noise control experiment.

With the determined control speaker and microphone configuration, the sound fields of the noise source and control speakers were measured in the anechoic chamber; a Wiener filter was obtained. Then, a global ANC experiment was conducted in each room using the obtained Wiener filter in an anechoic chamber. Figure 5 shows the schematic diagram of the global ANC experiment.

**Figure 6:**
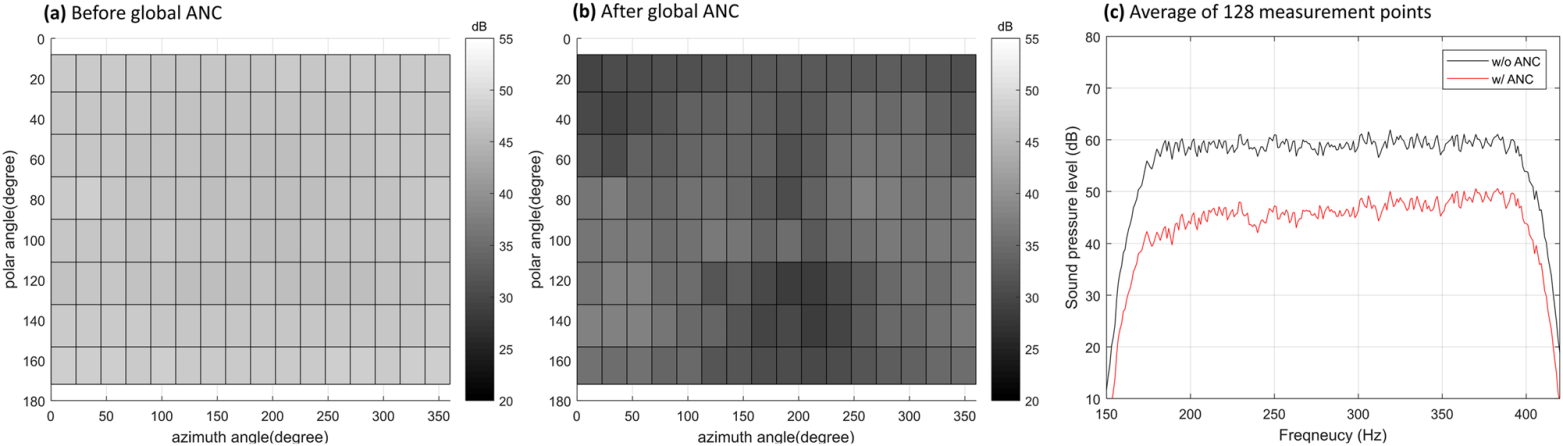
Global ANC experiment results at the anechoic chamber. (**a**) An average sound pressure level distribution of compound sound source in the frequency range of the interest before global ANC; (**b**) after global ANC; (**c**) Frequency domain result of an average of 128 measurement points.

First, with the calculated Wiener filter, the sound field before and after ANC was measured using the same 128 points in an anechoic chamber. Gaussian white noise filtered by the frequency range of interest was used as a reference signal. Figure 6 shows the average sound pressure level distribution of compound sound source (noise source and control speakers) in the frequency range of interest according to azimuth angle and polar angle and frequency domain results of an average of 128 measurement points.

**Figure 7:**
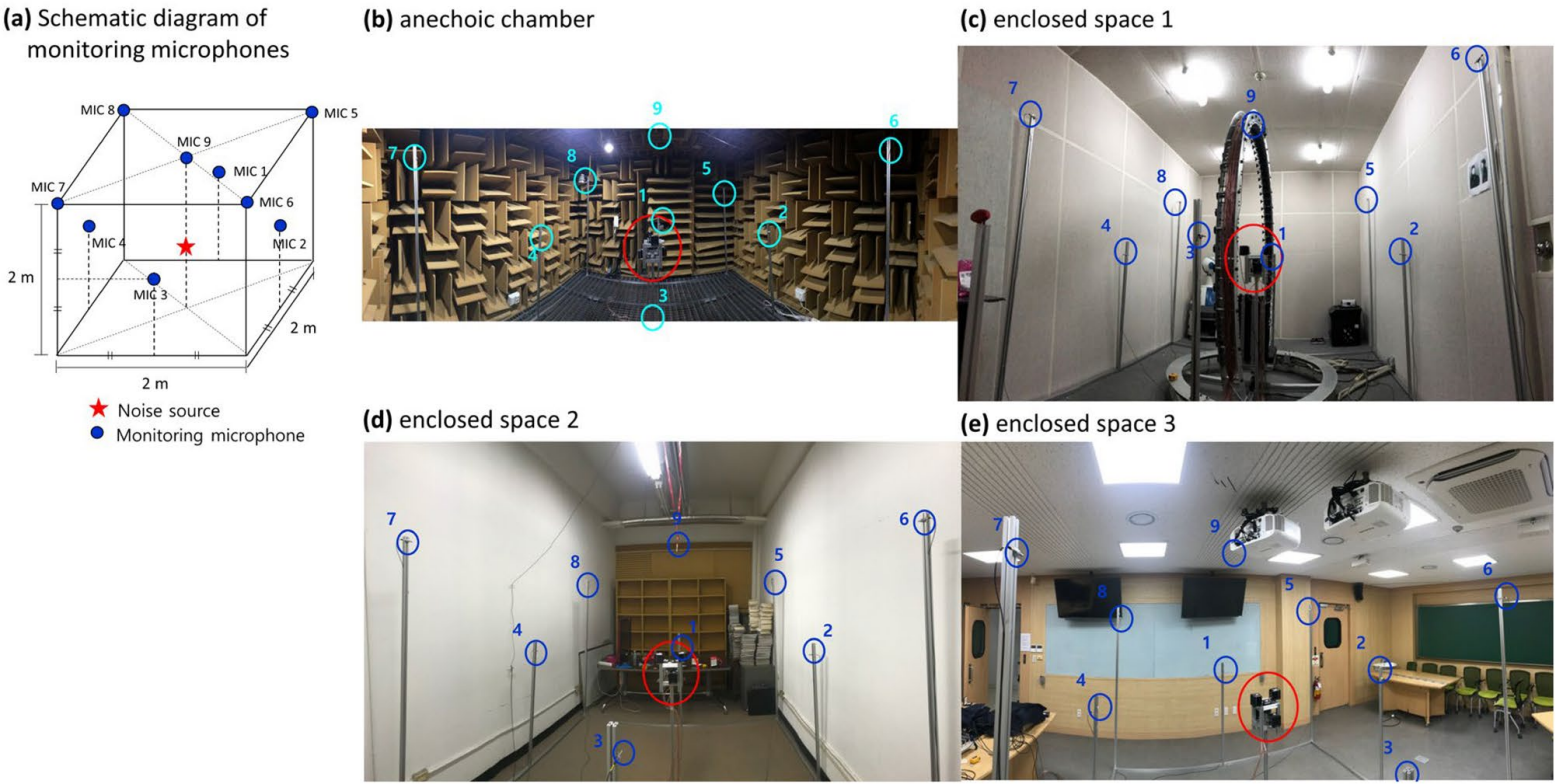
Schematic diagram of monitoring microphones and its experimental settings in each room. (**a**) Schematic diagram. (**b**) Anechoic chamber. (**c**) Enclosed space 1 (small size, small reverberation time). (**d**) Enclosed space 2 (small size, large reverberation time). (**e**) Enclosed space 3 (large size). Red circle is noise source and control speaker array. Blue circles are nine monitoring microphones for ANC control performance evaluation according to KS C IEC 60704-1: 2015.

Afterward, global ANC experiments in enclosed spaces were conducted to verify the performance of the controller designed in the free field. Global ANC was performed in the anechoic chamber and three enclosed spaces using the controller obtained in the anechoic chamber. The three enclosed spaces were selected to have different reverberation characteristics and were classified by reverberation time and relative size. The size of anechoic chamber was 3.6 $\times$ 3.6 $\times$ 2.4 ${\mathrm{m}}^{3}$. Enclosed space 1’s size was 3.2 $\times$ 5.5 $\times$ 2.8 ${\mathrm{m}}^{3}$; reverberation time was 0.26 s. Enclosed space 2’s size was 6.1 $\times$ 3.1 $\times$ 3.8 ${\mathrm{m}}^{3}$; reverberation time was 2.2 s. Enclosed space 3’s size was 6.6 $\times 9$.9 $\times$ 2.6 ${\mathrm{m}}^{3}$; reverberation time was 0.7 s. Figure 7 shows the positions of nine monitoring microphones for performance measurement placed near the noise source according to electronic products noise measurement specifications (KS C IEC 60704-1: 2015). This is the noise measurement standard to satisfy technical regulations for electrical and telecommunication products and components. Since we considered electronic products as potential applications for the proposed method, we tried to use their noise measurement standard.

**Figure 8:**
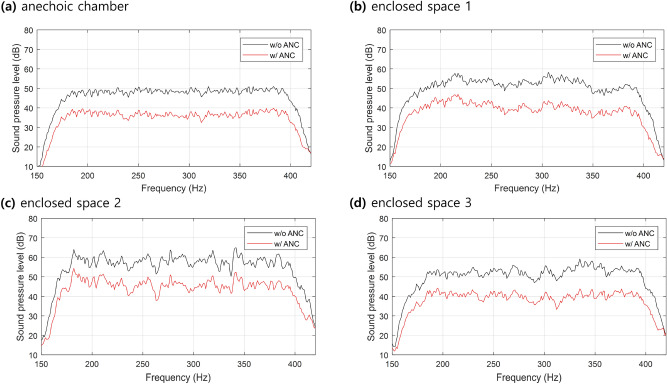
Frequency domain global ANC experiment results of an average of nine monitoring microphones. (**a**) Anechoic chamber; (**b**) Enclosed space 1 (small size, small reverberation time). (**c**) Enclosed space 2 (small size, large reverberation time). (**d**) Enclosed space 3 (large size). Black lines indicate noise before ANC, and red lines indicate noise after ANC.

Figure 8 shows the frequency domain global ANC results of an average of nine monitoring microphones for each space in the frequency range of interest, throughout the entirety of which noise has been reduced. Table 2 shows the average reduction in the frequency range of interest at each microphone and the maximum, minimum, and average at each space. The average reduction in the frequency range of interest is calculated as the below equation.

20 $$Average \ reduction=10{\mathrm{log}}_{10}\frac{{\int }_{F}{\left|{P}_{n}(f)\right|}^{2}df}{{\int }_{F}{\left|E(f)\right|}^{2}df}$$

where, ${P}_{n}$ is the sound pressure by noise source at frequency domain and $E$ is the sound pressure after control at frequency domain. $F$ is the frequency range of interest.

**Table 2:** Average reduction at each microphone in the frequency range of interest and their maximum, minimum, and average in each space. Nine microphones were placed according to KS C IEC 60704-1: 2015.

	Anechoic chamber	Enclosed space 1	Enclosed space 2	Enclosed space 3
mic 1	11.2 dB	12.1 dB	11.5 dB	11.0 dB
mic 2	11.2 dB	11.2 B	11.7 dB	11.9 dB
mic 3	11.6 dB	13.0 dB	11.8 dB	12.2 dB
mic 4	11.6 dB	10.3 dB	11.5 dB	11.7 dB
mic 5	11.1 dB	11.0 dB	11.6 dB	11.4 dB
mic 6	9.4 dB	11.0 dB	11.9 dB	11.1 dB
mic 7	12.4 dB	11.2 dB	11.3 dB	13.3 dB
mic 8	10.0 dB	11.2 dB	11.2 dB	11.3 dB
mic 9	15.6 dB	14.5 dB	12.5 dB	14.6 dB
Maximum	15.6 dB	14.5 dB	12.5 dB	14.6 dB
Minimum	9.4 dB	10.3 dB	11.2 dB	11.0 dB
Average	11.6 dB	11.8 dB	11.7 dB	12.3 dB

## Discussion

According to the results in Table. 1, it is shown that the reduction performance of the free field controller (proposed method) was higher than another controller except for point 1. This means that the enclosed space controller designed when the noise source is at point 1 performed worse than the free field controller at the other points in the enclosed space. Through the simulation results, it was found that the free field controller can achieve a satisfactory level of global reduction even in the enclosed space. Even compared to a controller designed in an enclosed space, the free field controller showed better performance if the noise source was located in a location different from where the enclosed space controller was calibrated. Therefore, we can predict that the free field controller will be effective as a suboptimal solution robust to the movement of noise sources.

From the results in Fig. 6, it can be confirmed that the magnitude of the sound pressure generated by the compound sound source is reduced for all directions through global ANC. This means that the acoustic power generated by the compound sound source has decreased. Since generated acoustic power is reduced, even if the compound sound source is placed in different enclosed spaces, global noise control is possible regardless of the noise source movement. As the result was measured in an anechoic chamber, the black line in Fig. 6c shows the frequency characteristics of the noise. The noise signal shows a flat frequency response in a frequency range of interest.

From the results in Fig. 8, after control, it can be observed that the overall magnitude of the noise is reduced, and the shape of the spectrum, which indicated the characteristics of the enclosed space, was evenly maintained. This means that global ANC reduced only the magnitude of the noise sound field, and it is consistent with the discussion about Fig. 6. Additionally, the Schroeder frequency of the enclosed space 1, 2, and 3 are 145 Hz, 350 Hz, and 128 Hz, respectively. Our frequency range of interest is from 170 to 378 Hz. So, in enclosed space 2, most of the frequency range of interest is under the Schroeder frequency, where the modal response is dominant. In the case of an anechoic chamber, it shows the flattest spectrum because it is not affected by the reflection field. Also, it can be seen that the frequency response of enclosed space 2 shows larger peaks and dips, which means modal response, compared to the relatively flat frequency responses of other enclosed spaces. The results of enclosed spaces 1 and 3 show that the proposed method is effective in the frequency range higher than the Schroeder frequency, unlike the existing global ANC method using modal characteristics. From the results in Table. 2, there were slight deviations from the maximum and minimum noise reduction levels, but the average values were similar. This shows that the controller designed in free field condition is effective even in other enclosed spaces.

## Conclusion

This study aimed to design a global ANC system that can be used in various acoustic environments. Conventional ANC systems for global ANC in an enclosed space require expensive and time-consuming experimental calibration: each time the acoustic environment changes, experimental calibration is required again. So, we designed a suboptimal open-loop controller in a free field that can be used in various acoustic environments. We proposed an experimental calibration approach for controller design in the free field. The arrangement of the control speakers and microphones was set to avoid spatial aliasing, which is determined according to the radiation pattern and frequency range of the noise source. Based on the configuration of the control speakers and microphones, the controller was designed to minimize the acoustic power in the free field. The simulation showed that a controller designed in the free field could achieve good performance despite the movement of the noise source. Based on these facts, a global ANC experiment was conducted. We conducted a global ANC experiment in other enclosed spaces using a controller designed in an anechoic chamber. Although the controller designed in an anechoic chamber was used, it shows good noise reduction performance in other enclosed spaces. These experimental results show that a controller designed in the free field can be effectively used in other enclosed spaces. Of course, in a stable acoustic environment, this suboptimal controller will show a slightly lower reduction performance than the optimal controller. However, an optimal controller in an enclosed space will require a large number of microphones for accurate calibration, and the performance will decrease if the acoustic environment changes. By designing a suboptimal controller in a free field, it will be possible to design a robust controller in a changing acoustic environment with a relatively simple experimental setup.

## Data Availability

The datasets generated and/or analyzed during the current study are available from the corresponding author on reasonable request.

## References

[1] Xiao T, Qiu X, Halkon B (2020). Ultra-broadband local active noise control with remote acoustic sensing. Sci. Rep..

[2] De Diego M, Gonzalez A (2001). Performance evaluation of multichannel adaptive algorithms for local active noise control. J. Sound Vib..

[3] Elliott SJ, Jung W, Cheer J (2018). Head tracking extends local active control of broadband sound to higher frequencies. Sci. Rep..

[4] Kwon B, Park Y (2013). Interior noise control with an active window system. Appl. Acoust..

[5] Lam B, Shi C, Shi D, Gan WS (2018). Active control of sound through full-sized open windows. Build. Environ..

[6] Murao T, Shi C, Gan WS, Nishimura M (2017). Mixed-error approach for multi-channel active noise control of open windows. Appl. Acoust..

[7] Nelson PA, Curtis ARD, Elliott SJ, Bullmore AJ (1987). The active minimization of harmonic enclosed sound fields, Part I: Theory. J. Sound Vib..

[8] Nelson PA, Curtis ARD, Elliott SJ, Bullmore AJ (1987). The active minimization of harmonic enclosed sound fields, part II: A computer simulation. J. Sound Vib..

[9] Nelson PA, Curtis ARD, Elliott SJ, Bullmore AJ (1987). The active minimization of harmonic enclosed sound fields, Part III: Experimental verification. J. Sound Vib..

[10] Sundararajan N, Williams JP, Montgomery RC (1985). Adaptive modal control of structural dynamic systems using recursivelattice filters. J. Guid. Control. Dyn..

[11] Montazeri A, Poshtan J, Kahaei MH (2007). Modal analysis for global control of broadband noise in a rectangular enclosure. J. Low Freq. Noise Vib. Active Control.

[12] Puri A, Modak SV, Gupta K (2018). Modal filtered-x LMS algorithm for global active noise control in a vibro-acoustic cavity. Mech. Syst. Signal Process..

[13] Puri A, Modak SV, Gupta K (2019). Global active control of harmonic noise in a vibro-acoustic cavity using Modal FxLMS algorithm. Appl. Acoust..

[14] Laugesen S, Elliott SJ (1993). Multichannel active control of random noise in a small reverberant room. IEEE Trans. Speech Audio Process..

[15] Joplin PM, Nelson PA (1990). Active control of low-frequency random sound in enclosures. J. Acoust. Soc. Am..

[16] Ciesielka W, Gołaś A (2006). An adaptive, active noise reduction system in closed space. Arch. Acoust..

[17] Maa DY (1994). Sound field in a room and its active noise control. Appl. Acoust..

[18] Schroeder MR, Kuttruff KH (1962). On frequency response curves in rooms. Comparison of experimental, theoretical, and Monte Carlo results for the average frequency spacing between maxima. J. Acoust. Soc. Am..

[19] Guo J, Hodgson M (1999). Active noise control in enclosed spaces. Can. Acoust..

[20] Li D, Hodgson M (2005). Optimal active noise control in large rooms using a “locally global” control strategy. J. Acoust. Soc. Am..

[21] Aslan F, Paurobally R (2018). Modelling and simulation of active noise control in a small room. J. Vib. Control.

[22] Zhang J, Zhang W, Zhang JA, Abhayapala TD, Zhang L (2021). Spatial active noise control in rooms using higher order sources. IEEE/ACM Trans. Audio Speech Lang. Process..

[23] Pierce AD (2019). Acoustics: an introduction to its physical principles and applications.

[24] Kempton AJ (1976). The ambiguity of acoustic sources: A possibility for active control?. J. Sound Vib..

[25] Ffowcs Williams JE (1984). Review lecture-anti-sound. Proc. R. Soc. Lond. A..

[26] Jessel MJM, Angevine OL (1980). Active acoustic attenuation of a complex noise source. Inter-noise 80.

[27] Jessel Maurice JM (1983). Active noise reduction as an experimental application of the general system theory. INTER-NOISE NOISE-CON Congr. Conf. Proc..

[28] Ross CF (1978). Experiments on the active control of transformer noise. J. Sound Vib..

[29] Piraux J, Nayroles B (1980). A theoretical model for active noise attenuation in three-dimensional space. INTER-NOISE NOISE-CON Congr. Conf. Proc..

[30] Angevine OL (1981). Active acoustic attenuation of electric transformer noise. J. Acoust. Soc. Am..

[31] Angevine OL (1983). Active acoustic absorption: Where does the energy go?. J. Acous. Soc. Am..

[32] Mangiante GA (1977). Active sound absorption. J. Acoust. Soc. Am..

[33] Ross CF (1981). A demonstration of active control of broadband sound. J. Sound Vib..

[34] Guo J, Pan J, Bao C (1997). Actively created quiet zones by multiple control sources in free space. J. Acous. Soc. Am..

[35] Nelson PA, Elliott SJ (1986). The minimum power output of a pair of free field monopole sources. J. Sound Vib..

[36] Nelson PA, Curtis ARD, Elliott SJ, Bullmore AJ (1987). The minimum power output of free field point sources and the active control of sound. J. Sound Vib..

[37] Mazur K, Wrona S, Pawelczyk M (2018). Design and implementation of multichannel global active structural acoustic control for a device casing. Mech. Syst. Signal Process..

[38] Chraponska A, Wrona S, Rzepecki J, Mazur K, Pawelczyk M (2019). Active structural acoustic control of an active casing placed in a corner. Appl. Sci..

[39] Wrona S, Pawelczyk M (2019). Feedforward control of double-panel casing for active reduction of device noise. J. Low Freq. Noise Vib. Active Control.

[40] Mazur K, Wrona S, Pawelczyk M (2020). Performance evaluation of active noise control for a real device casing. Appl. Sci..

[41] Dmochowski J, Benesty J, Affès S (2008). On spatial aliasing in microphone arrays. IEEE Trans. Signal Process..

[42] Otani M, Watabe H, Tsuchiya T, Iwaya Y (2017). Effects of spatial aliasing in sound field reproduction: Reproducibility of binaural signals. Acoust. Sci. Technol..

[43] Rafaely B (2018). Fundamentals of Spherical Array Processing.

[44] Nettingsmeier, J. Higher order Ambisonics-a future-proof 3D audio technique. *Verband Deutscher Tonmeister International Convention* (2010).

[45] Rupp K, Jungemann C, Hong SM, Bina M, Grasser T, Jüngel A (2016). A review of recent advances in the spherical harmonics expansion method for semiconductor device simulation. J. Comput. Electron..

[46] Gupta A, Abhayapala TD (2010). Three-dimensional sound field reproduction using multiple circular loudspeaker arrays. IEEE Trans. Audio Speech Lang. Process..

[47] Pasqual, A. M. *Sound Directivity Control in a 3-D Space by a Compact Spherical Loudspeaker Array* (Doctoral dissertation) (2010). 10.1121/1.3500689 21218880

[48] Kuntz, A. & Rabenstein, R. An approach to global noise control by wave field synthesis. *2004 12th European Signal Processing Conference* 1999–2002 (2004).

[49] Kuo SM, Morgan DR (1996). Active Noise Control Systems: Algorithms and DSP Implementations.

[50] Zotter F, Frank M (2019). Ambisonics: A Practical 3D Audio Theory for Recording, Studio Production, Sound Reinforcement, and Virtual Reality.

[51] Hayes MH (2009). Statistical Digital Signal Processing and Modeling.

